# Endoscopically-detected small-intestinal lesions with dense infiltration of IgG4-positive plasma-cells

**DOI:** 10.1007/s12328-026-02312-5

**Published:** 2026-03-27

**Authors:** Hiroki Goto, Yu Sasaki, Yasuhiko Abe, Makoto Yagi, Naoko Mizumoto, Yusuke Onozato, Minami Ito, Yoshiyuki Ueno

**Affiliations:** 1 Department of Gastroenterology, Faculty of Medicine, Yamagata University, 2-2-2 Iida-Nishi, Yamagata, 990-9585 Japan; 2 Division of Endoscopy, Yamagata University Hospital, 2-2-2 Iida-Nishi, Yamagata, 990-9585 Japan

**Keywords:** IgG4-RD, Small intestinal ulcer, Intestinal stricture

## Abstract

Immunoglobulin G4-related disease (IgG4-RD) is a fibroinflammatory syndrome characterized by dense IgG4-positive plasma-cell infiltrate and progressive fibrosis. Small intestinal involvement is uncommon, and its clinicopathological spectrum remains unclear. Herein, we describe two older individuals with small intestinal lesions rich in IgG4-positive plasma cells. Case 1 was a 73-year-old woman with known orbital and pancreatic IgG4-RD who developed mild ileal narrowing, detected on surveillance imaging, and elevated serum IgG4 (214 mg/dL). Case 2 was an 87-year-old woman without prior IgG4-RD who presented with bowel obstruction caused by a tight ileal stricture, normal serum IgG4 level (59 mg/dL), and no other organs affected. In both patients, histological examination revealed dense lymphoplasmacytic infiltration, with IgG4/IgG ratios exceeding 40%. Although hyper-IgG4-emia and multiorgan involvement commonly trigger diagnostic suspicion, these cases demonstrate that IgG4-rich inflammation can arise as a solitary manifestation in the small intestine, and may escape detection unless specific immunostaining is conducted. Sequential organ involvement is also possible, underscoring the need for longitudinal follow-up. IgG4‑positive plasma‑cell infiltration should be included in the differential diagnosis of unexplained small‑intestinal ulcers or strictures—even when serum IgG4 is normal and systemic features of IgG4‑RD are absent—as histological confirmation can guide timely immunosuppressive or surgical management.

## Introduction

Immunoglobulin G4-related disease (IgG4-RD) is a chronic systemic fibroinflammatory disorder characterized by dense infiltration of IgG4-positive plasma cells, commonly accompanied by elevated serum IgG4 concentrations and progressive, potentially organ-impairing fibrosis [1]. IgG4-RD can affect a broad spectrum of sites, including the pancreas, biliary tree, salivary and lacrimal glands, thyroid gland, lymph nodes, retroperitoneum, kidneys, and skin; however, primary small bowel involvement is exceedingly uncommon [2]. Indeed, the current European guidelines for IgG4-related digestive diseases state that small intestinal diseases are “extremely rare or absent” [3]. Most published reports describe IgG4-related small intestinal lesions only indirectly in the context of postoperative diagnoses of sclerosing mesenteritis; however, a few well-documented cases of IgG4-related enteritis have been reported [4, 5].

Herein, we describe two exceptional cases in which small intestinal lesions rich in IgG4-positive plasma cells were detected endoscopically. One patient exhibited enteric disease as a manifestation of systemic IgG4-RD, whereas the other developed severe, localized jejunal stricture without any elevation in serum IgG4 levels or extraintestinal involvement.

These cases highlight several important factors. First, hyper-IgG4-emia or multiorgan disease usually prompts consideration of IgG4-RD; in their absence, the diagnosis may be missed if histopathology is not specifically evaluated using IgG4 immunostaining. Second, these cases suggest the importance of including IgG4-positive plasma-cell infiltration in the differential diagnosis of unexplained small-intestinal lesions, even when serum IgG4 levels are normal, and no other organs appear to be affected. Both patients provided informed consent for publication of this report.

## Case reports

### Case 1

**Fig. 1:**
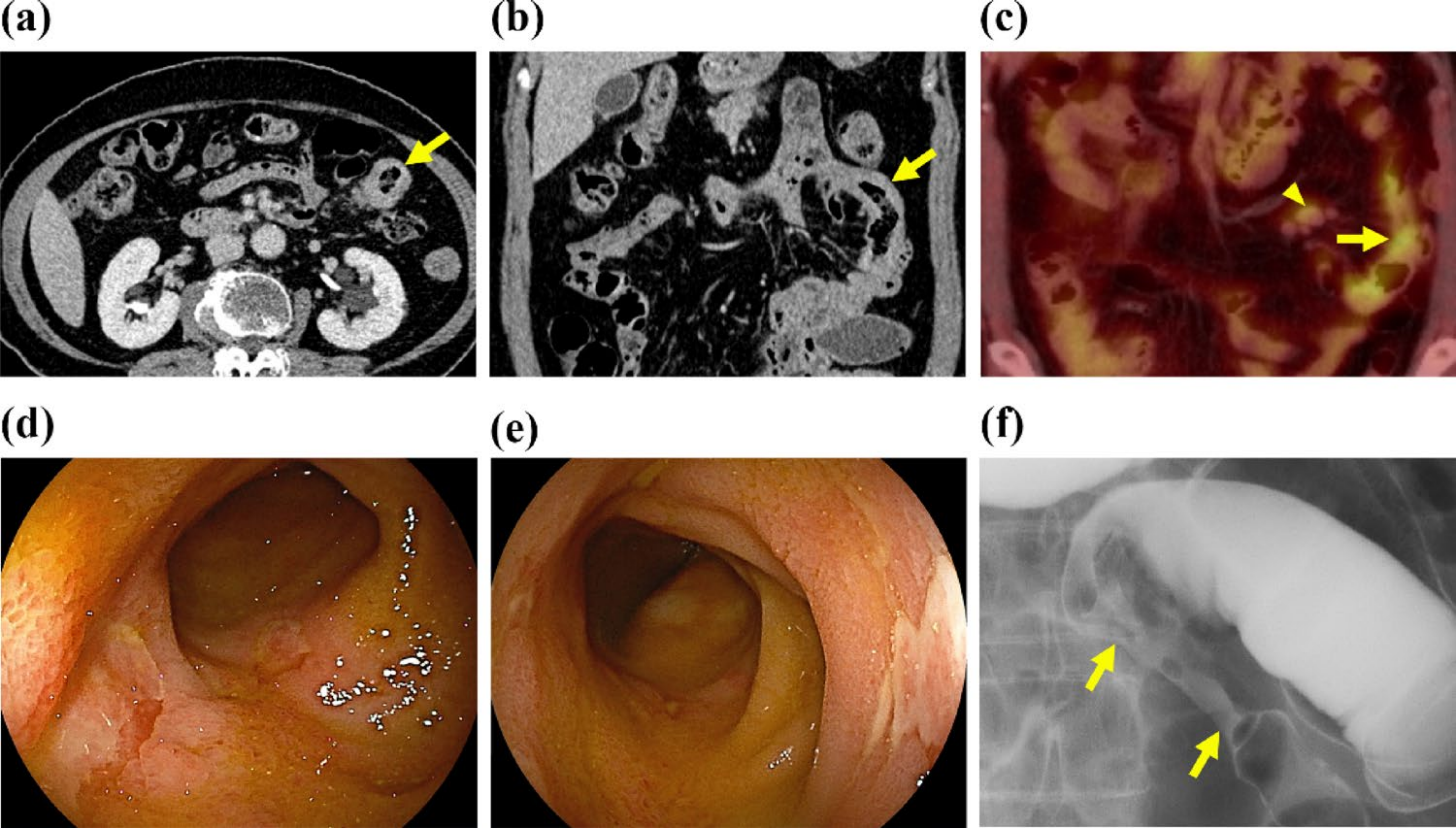
Radiological and endoscopic images of the small intestinal lesion in Case 1. **a**, **b** Axial and coronal enhanced computed tomography images showing thickening of the proximal jejunal wall with luminal narrowing, upstream dilatation (yellow arrows). **c** FDG PET/CT images showing intense FDG uptake in both the thickened jejunal segment (SUVmean = 10.0; SUVmax = 11.6, yellow arrow) and the enlarged lymph nodes (SUVmean = 7.4; SUVmax = 8.8, yellow arrowheads). **d**, **e** Antegrade double-balloon enteroscopy (EN-580T; Fujifilm, Tokyo, Japan) reveals multiple shallow ulcers in the ileum. **f** Contrast injection during enteroscopy delineates a mild 40-mm-long luminal stenosis (yellow arrows)

**Fig. 2:**
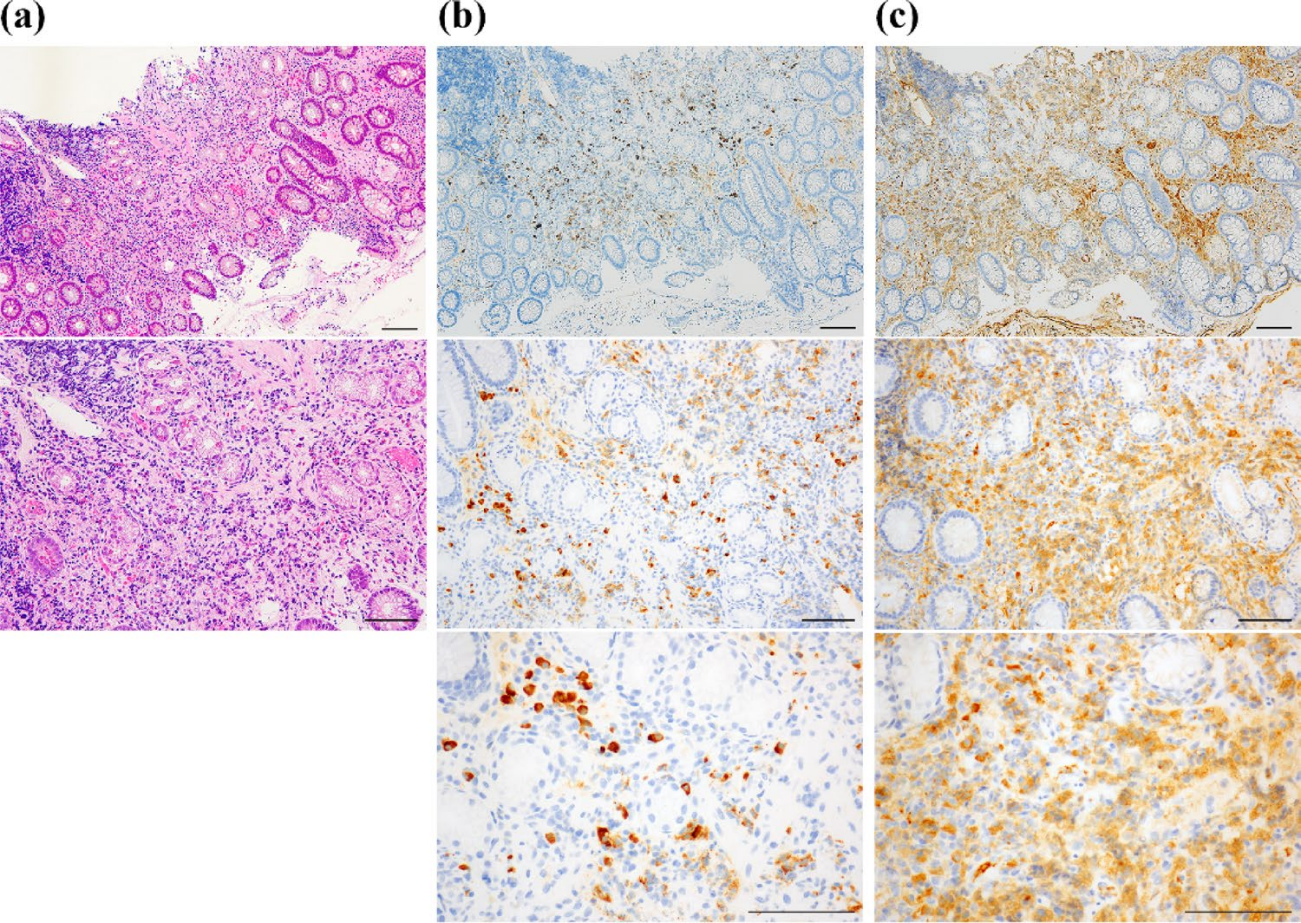
Pathological findings of the endoscopic biopsied specimens in Case 1. **a** Hematoxylin**–**eosin staining showing dense lymphoplasmacytic infiltration. **b** Immunostaining for IgG4 highlights > 50 IgG4-positive plasma cells/HPF. **c** Total IgG immunostaining in the same field; the IgG4/ IgG ratio exceeds 50%. The bar line corresponds to 100 μm

Case 1 was a 73-year-old woman who was referred to our hospital after focal thickening of the small bowel wall was noted on abdominal computed tomography (CT). She had a history of IgG4-positive orbital pseudotumors 12 years prior and autoimmune pancreatitis (AIP) 10 years prior, both of which were managed conservatively because she remained asymptomatic and her serum IgG4 concentrations were within the reference range. She did not present with any symptoms. Physical examination findings were unremarkable, and no salivary, thyroid, or retroperitoneal involvement was noted. She had no recent history of nonsteroidal anti-inflammatory drug (NSAID) use. Laboratory tests revealed normal levels of IgG (1,364 mg/dL), carcinoembryonic antigen (CEA; 4.13 µg/L), carbohydrate antigen 19-9 (CA19-9; 21.4 kU/L), and soluble interleukin-2 receptor (sIL-2R; 647 U/mL); however, her serum IgG4 level was elevated at 214 mg/dL. Contrast-enhanced CT revealed eccentric thickening of the left ileal wall (Fig. 1a, b). Fluorodeoxyglucose positron emission tomography/computed tomography (FDG PET/CT) revealed intense FDG uptake in both the thickened small intestinal wall and swollen lymph nodes (Fig. 1c). Antegrade double-balloon enteroscopy revealed multiple shallow ulcers and a 40-mm segment of mild luminal narrowing in the ileum (Fig. 1d–f). An ulcer biopsy revealed dense lymphoplasmacytic infiltration. More than 50 IgG4-positive plasma cells were present per high-power field (HPF), and the IgG4/IgG ratio exceeded 50% (Fig. 2). Storiform fibrosis and obliterative phlebitis were not identified; however, the assessment of these features is inherently limited in biopsy specimens. Esophagogastroduodenoscopy, colonoscopy, double-balloon enteroscopy, contrast-enhanced CT, and FDG PET/CT showed no additional lesions elsewhere in the gastrointestinal tract. The differential diagnosis included small-bowel neoplasms, Crohn’s disease, and NSAID-induced enteropathy. Neoplasia was deemed unlikely because imaging revealed no discrete mass or invasive features, and biopsies showed no dysplasia or malignant cytology. Crohn’s disease was not supported by the clinical, endoscopic, or radiologic findings. No noncaseating epithelioid granulomas were identified. NSAID-related injury was unlikely given the absence of recent NSAID exposure. These findings satisfied the comprehensive diagnostic criteria for IgG4-RD with small-intestinal involvement (2019 American College of Rheumatology/European League Against Rheumatism [6]: ACR/EULAR score 36). Prednisolone therapy was advised, but the patient elected to undergo only observation. She remained asymptomatic during 6 months of follow-up at our institution and subsequently returned to her referring hospital. More than 5 years have elapsed since detection of the ileal lesion, and no ileal lesion-related adverse events have been reported to date.

## Case 2

**Fig. 3:**
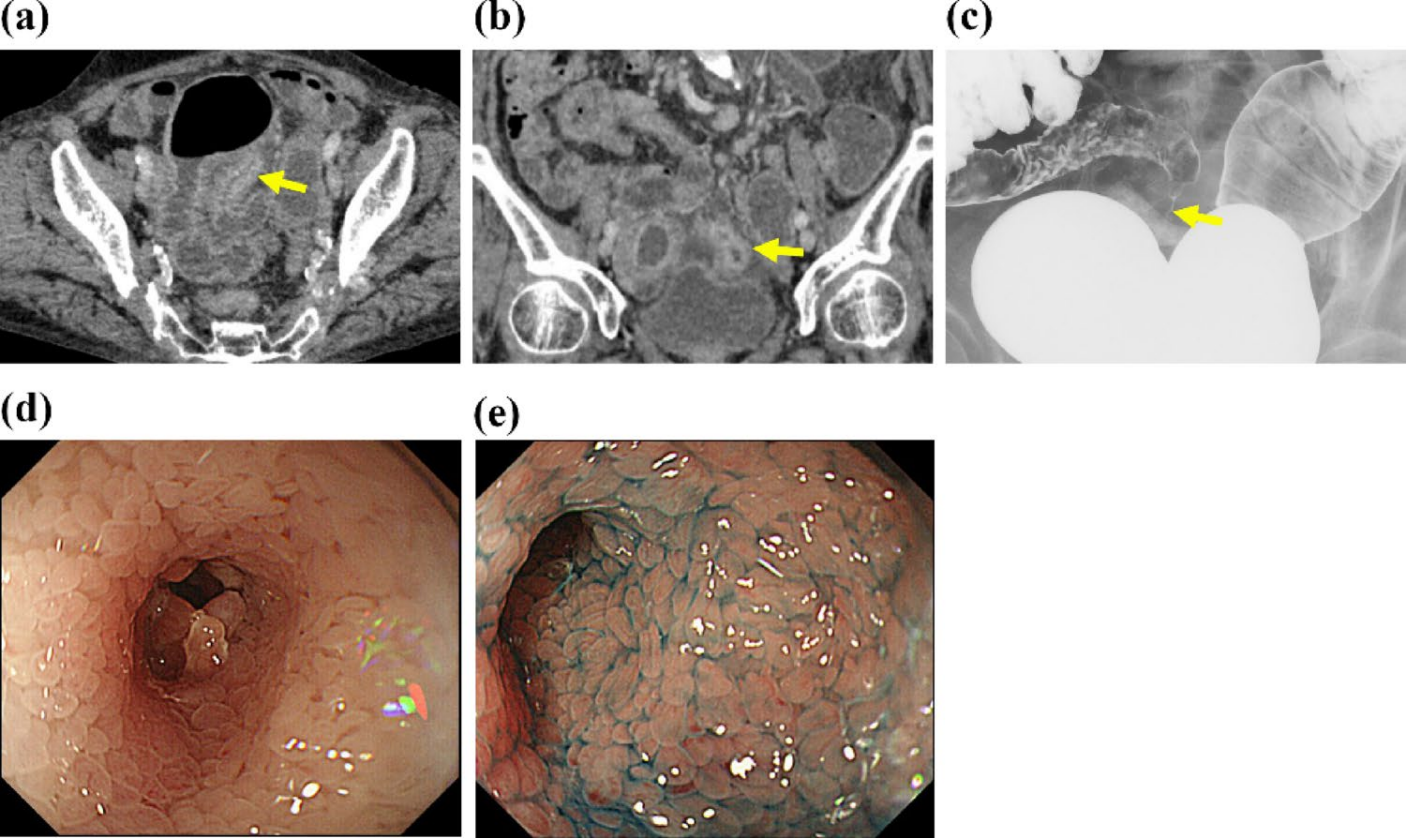
Radiological and endoscopic images of the small intestinal lesion in Case 2. **a**, **b** Contrast-enhanced CT showing a focally thickened ileum (yellow arrows). **c** Small-bowel contrast study demonstrating a discrete short-segment stricture (yellow arrow). **d**, **e** Colonoscopy showing a subepithelial tumor-like mass proximal to the ileocecal valve, producing high-grade luminal narrowing

**Fig. 4:**
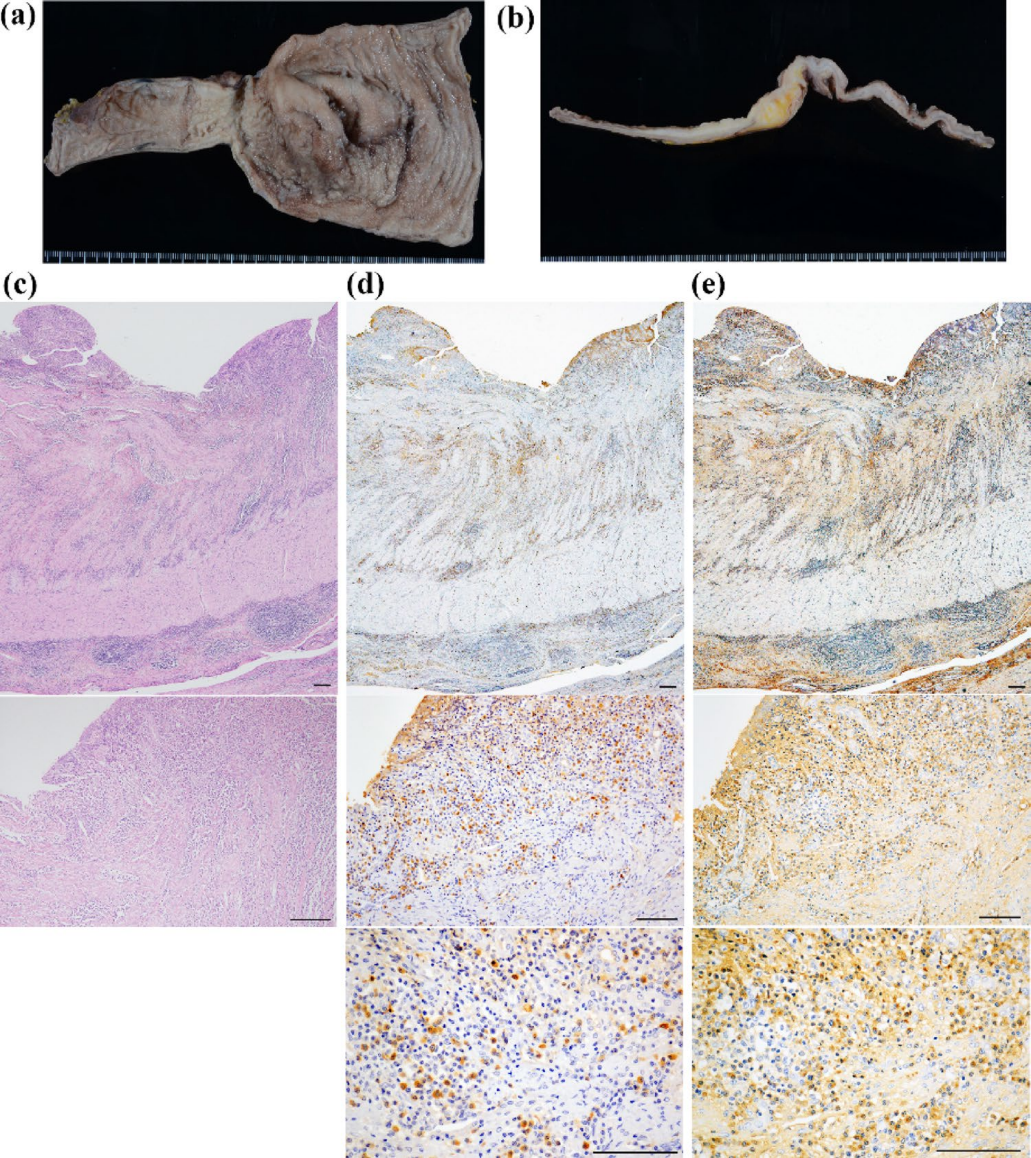
Pathological findings of the surgical resected specimen in Case 2. Images depict the surgically resected small intestinal lesion (**a**) and sectional (**b**) levels. **c** Hematoxylin**–**eosin staining confirms transmural lymphoplasmacytic infiltration. **d** IgG4 immunostaining demonstrated > 50 IgG4-positive plasma cells per HPF. **e** Total IgG immunostaining of the corresponding areas; the IgG4/ IgG ratio exceeds 40%. The bar represents 100 μm

An 87-year-old woman presented with a 2-month history of anorexia. Eight months earlier, she had experienced an episode of small bowel obstruction that resolved with conservative care. CT performed at the referring hospital revealed small bowel dilatation, prompting transfer for suspected recurrent obstruction. No lesions were detected in the salivary glands, thyroid glands, or retroperitoneum. Laboratory findings revealed normal levels of IgG4 (59 mg/dL), CEA (1.07 µg/L), CA19-9 (< 2.0 kU/L), and sIL2-R (878 U/mL), but elevated IgG (2,650 mg/dL). CT performed at our institution revealed focal thickening of the ileum (Fig. 3a, b). A small bowel contrast study revealed a discrete stricture (Fig. 3c), whereas ileocolonoscopy revealed severe luminal stenosis approximately 20 cm proximal to the ileocecal valve (Fig. 3d, e). However, endoscopic passage was not possible. Upper gastrointestinal endoscopy showed no abnormal findings. Capsule endoscopy and double-balloon enteroscopy were not performed given the severe ileal stricture and associated risk of obstruction. The small-bowel contrast study showed no additional lesions apart from the ileal stricture. Endoscopic biopsies and subsequent laparoscopic segmental ileal resection revealed marked lymphoplasmacytic infiltration (Fig. 4a–c). More than 50 IgG4-positive plasma cells/HPF were identified, and the IgG4/IgG ratio was > 40% (Fig. 4d, e). Storiform fibrosis and obliterative phlebitis were not observed, and there was no thickening of the intrinsic muscular layer or bottom-heavy plasmacytosis. No noncaseating epithelioid granulomas were identified, and there were no fissuring ulcers or transmural inflammation changes suggestive of Crohn’s disease. Endoscopic and imaging examinations showed no additional lesions elsewhere in the gastrointestinal tract. Differential diagnoses, including neoplasia, Crohn’s disease, and NSAID-induced enteropathy, were excluded based on the patient’s history and pathology results. The patient was ultimately diagnosed with a localized IgG4-rich ileal stricture causing obstruction, best considered an organ-limited IgG4-risk lesion with uncertain assignment to IgG4-RD (ACR/EULAR score 18). IgG subclasses other than IgG4 and postoperative immunoglobulin levels were not obtained. Symptoms resolved after surgery, and the postoperative course was uneventful. After disclosure of the pathological diagnosis, follow-up at our institution was concluded at the patient’s request, given her advanced age.

## Discussion

The concept of AIP was first proposed by Yoshida et al. in 1995, in a report describing several cases of chronic pancreatitis characterized by steroid responsiveness, dense lymphoplasmacytic infiltration, and frequent positivity for antinuclear antibodies [7]. In 2001, Hamano et al. demonstrated that rich IgG4-positive plasma cell infiltration and fibrosis could occur not only in the pancreas, but also in extrapancreatic organs, accompanied by elevated serum IgG4 levels [8]. Two years later, Kamisawa et al. proposed the unifying designation “IgG4-RD,” encompassing a wide spectrum of organ manifestations [9].

Although many extrapancreatic sites have since been documented, the 2020 European guidelines on IgG4-related digestive diseases characterize small bowel involvement as extremely rare or absent, and provide no disease-specific diagnostic criteria [3]. Therefore, these two cases included an uncommon clinical subset. Case 1 fulfilled the 2019 ACR/EULAR classification criteria for IgG4-RD, with a score of 36 owing to prior orbital and pancreatic disease and elevated serum IgG4, which is well above the 20‑point threshold [8]. In contrast, Case 2 scored only 18, as her serum IgG4 level was normal and no other organs were involved. Accordingly, Case 1 is classified as IgG4-RD with small-intestinal involvement (ACR/EULAR score 36), whereas Case 2 is best described as an organ-limited IgG4-rich ileal lesion with uncertain assignment to IgG4-RD (score 18). Small intestinal IgG4-RD lesions without involvement of other organs have previously been reported, with studies suggesting that organ involvement in IgG4-RD may occur sequentially, rather than synchronously [10]. Additionally, isolated small intestinal IgG4-positive plasma cell-rich lesions without hyper-IgG4-emia have been described following the exclusion of inflammatory bowel disease (IBD) and sclerosing mesenteritis [11–13]. Thus, the ileal stricture in Case 2 could represent either an early, organ-limited manifestation of IgG4-RD or an IgG4-rich inflammatory lesion unrelated to IgG4-RD. Although there have been several reports of long-term remission without recurrence for approximately 5 years following the resection of small intestinal lesions, careful long-term surveillance is warranted not only for the potential recurrence of intestinal lesions, but also for the emergence of IgG4-related manifestations in other organs [12].

Although the hallmark features of storiform fibrosis and obliterative phlebitis were not identified—likely influenced by sampling [14] in Case 1 or the context of organ/stage variability [15] in Case 2—both lesions satisfied quantitative histologic thresholds that warrant suspicion for IgG4-related disease (> 50 IgG4+ cells/HPF and IgG4/IgG ≥ 40%). Accordingly, our final classification was made by clinicopathologic integration, taking into account serum IgG4 levels and the absence of extraintestinal involvement.

Solitary gastrointestinal IgG4-RD commonly mimics malignancy, and preoperative diagnosis is often challenging. Most published cases have been identified only after resection, with serum IgG4 frequently going unmeasured [11, 16]. If recognized endoscopically, steroid therapy may obviate surgery. However, when a neoplasm cannot be definitively excluded, surgical resection remains prudent [17].

Only four cases, including the present patient (Table 1), have been reported, in which small intestinal lesions with rich infiltration of IgG4-positive plasma cells were diagnosed endoscopically [4, 5]. Serum IgG4 levels were elevated in three patients, all of whom had multiorgan disease, supporting a diagnosis of true IgG4-related enteritis. Reported endoscopic findings vary and include multiple shallow ulcers, longitudinal ulcers, and strictures; therefore, the characteristic features have not yet been defined.

**Table 1:** Characteristics of the present and previously reported cases of small intestinal lesions with IgG4-positive plasma cell infiltration identified using endoscopy

Author	Age/Sex	Symptom	Location	Endoscopic findings	Extraluminal lesion	Serum IgG4 (mg/dL)	IgG4/IgG ratio (%)	Treatment	Prognosis
Fujita et al. [5]	55/M	Abdominal pain, fatigue	Jejunum	Multiple ulcers	Autoimmune pancreatitis	2,480	> 70	Oral prednisolone	No recurrence
Yoshidome et al. [4]	70/M	Abdominal pain, fever, headache	Ileum	Longitudinal ulcer	Hypertrophic pachymeningitis	393	> 50	Steroid-pulse therapy	No recurrence
Present case 1	73/F	None	Ileum	Multiple ulcers, stenosis	Autoimmune pancreatitis dacryoadenitis	214	> 50	Follow up	No obstruction symptoms
Present case 2	87/F	Abdominal pain	Ileum	Multiple ulcers, stenosis	None	59	> 40	Partial small bowel resection	No recurrence

HPF, high-power field

It is important to note that IgG4-positive plasma cell infiltration is not specific to IgG4-related disease. Several gastrointestinal disorders have been reported to show increased IgG4-positive plasma cells. IgG4-positive plasma cell infiltration can be observed in IBD, particularly in patients with ulcerative colitis. IgG4-positive plasma cell infiltration has also been reported in Crohn’s disease, although at a lower frequency than in ulcerative colitis [18, 19]. Thus, this finding alone may not be pathognomonic [20]. Among the 119 colonic biopsies from patients with IBD without AIP, 17.6% contained > 10 IgG4-positive plasma cells/HPF, and 4.2% had elevated serum IgG4 levels [21]. In Case 2, the absence of IBD-related clinical, endoscopic, and histological features argues that IgG4-positive plasma cells constituted the primary pathology, rather than a secondary response.

In addition to IBD, prominent IgG4-positive plasma-cell infiltrates have been reported in pediatric eosinophilic esophagitis, with 48% (15/31) of biopsies showing increased IgG4-positive cells [22]. In celiac disease, > 10 IgG4-positive plasma cells/HPF were present in 39% (7/18) duodenal biopsies, and increased IgG4-positive cells were identified in 21.4% (3/14) non-celiac controls [23]. Furthermore, autoimmune gastritis has been associated with increased IgG4-positive plasma cells in 37% of patients [24]. Therefore, final classification requires clinicopathological integration, including clinical context, endoscopic findings, architectural features, serology, and systemic evaluation, rather than reliance on IgG4 staining alone.

Some small-bowel lesions previously labelled “idiopathic” may in fact be IgG4-related. When evaluating unexplained ulcers or strictures endoscopically, clinicians should include IgG4-RD in the differential diagnosis, even if serum IgG4 is normal and no systemic disease is evident, and should consider requesting immunostaining for IgG4 in biopsy specimens. Early recognition permits timely immunosuppressive therapy and may help to avoid unnecessary surgeries.

In conclusion, accumulating evidence indicates that IgG4-RD can present as a small-intestinal disease, sometimes without hyper-IgG4-emia. Routine consideration of IgG4 immunostaining in unexplained small-bowel ulcers or strictures may increase diagnostic yield, facilitate timely immunosuppressive therapy, and refine our understanding of this evolving entity. Ongoing multicenter registries and longitudinal studies are required to define the incidence, natural history, and optimal management strategies of small-intestinal IgG4-RD.
